# Development and Validation of a Personalized, Web-Based Decision Aid for Lung Cancer Screening Using Mixed Methods: A Study Protocol

**DOI:** 10.2196/resprot.4039

**Published:** 2014-12-19

**Authors:** Yan Kwan Lau, Tanner J Caverly, Sarah T Cherng, Pianpian Cao, Mindy West, Douglas Arenberg, Rafael Meza

**Affiliations:** ^1^Department of EpidemiologySchool of Public HealthUniversity of MichiganAnn Arbor, MIUnited States; ^2^Division of General Internal MedicineDepartment of Internal MedicineUniversity of Michigan Medical SchoolAnn Arbor, MIUnited States; ^3^Center for Clinical Management ResearchVeterans Affairs Ann Arbor Healthcare SystemAnn Arbor, MIUnited States; ^4^Division of Pulmonary & Critical MedicineDepartment of Internal MedicineUniversity of Michigan Medical SchoolAnn Arbor, MIUnited States

**Keywords:** informed decision-making, lung cancer screening, patient decision aid, patient education

## Abstract

**Background:**

The National Lung Screening Trial demonstrated that low-dose computed tomography (LDCT) screening could be an effective way to reduce lung cancer mortality. Informed decision-making in the context of lung cancer screening requires that potential screening subjects accurately recognize their own lung cancer risk, as well as the harms and benefits associated with screening, while taking into account their personal values and preferences.

**Objective:**

Our objective is to develop a Web-based decision aid in accordance with the qualifying and certification criteria in the International Patient Decision Aid Standards instrument version 4.0 that will assist patients in making informed decisions with regard to lung cancer screening.

**Methods:**

In “alpha” testing, a prototype of the decision aid was tested for usability with 10 potential screening participants in focus groups. Feedback was also sought from public health and health risk communication experts external to the study. Following that, improvements to the prototype were made accordingly, and “beta” testing was done in the form of a quasi-experimental design—a before-after study—with a group of 60 participants. Outcomes tested were knowledge, risk perception of lung cancer and lung cancer screening, decisional conflict, and acceptability of the decision aid as determined by means of a self-administered electronic survey. Focus groups of a subsample of survey participants will be conducted to gain further insight into usability issues.

**Results:**

Alpha testing is completed. Beta testing is currently being carried out. As of 2014 December 7, 60 participants had completed the before-after study. We expect to have results by 2015 January 31. Qualitative data collection and analysis are expected to be completed by 2015 May 31.

**Conclusions:**

We hypothesize that this Web-based, interactive decision aid containing personalized, graphical, and contextual information on the benefits and harms of LDCT screening will increase knowledge, reduce decisional conflict, and improve concordance between patient preferences and the current US Preventive Services Task Force’s screening guidelines.

## Introduction

Lung cancer remains the leading cause of cancer death in the United States [1]. The National Lung Screening Trial demonstrated that lung cancer screening with low-dose computed tomography (LDCT) has the potential to significantly reduce lung cancer mortality [2]. On the basis of this and other evidence, the US Preventive Services Task Force (USPSTF) gave a B recommendation for LDCT screening [3,4], the same strength of recommendation associated with mammography screening. However, real-world success in lung cancer screening will be conditional on identifying and screening those at highest risk for lung cancer while discouraging screening in those at low risk. Lung cancer screening presents a challenge, because it is the first population-wide screening modality with eligibility criteria based not only on age but also on a lifestyle behavior (at least 30 pack-years of tobacco use and smoking within the past 15 years). Identifying those at risk and helping them understand the benefits of screening and how to reduce their risk (e.g., tobacco cessation) is paramount for an effective implementation of population-wide lung cancer screening.

Implementing lung cancer screening in an environment where patients do not have the tools or information to understand disease risks and the harm-benefit balance of screening will most likely be counterproductive. In addition to providing information for individuals regarding lung cancer screening that allows them to weigh the potential harms of LDCT in accordance with the benefits, we also recognize that the decision to be screened is preference-sensitive. In light of this, there is a need to assist individuals with making informed decisions regarding lung cancer screening in which their personal values are also taken into account.

The USPSTF defines informed decision-making as “an individual’s overall process of gathering relevant health information from both his or her clinician and from other clinical and nonclinical sources, with or without independent clarification of values” ( [5], p. 59). In particular, a patient decision aid’s functions are (1) to provide facts about an individual’s condition and the options available and their characteristics, (2) to help individuals clarify values and personal preferences, and (3) to assist these individuals to discuss their values and preferences with health professionals [6]. It is in this context that our goal is defined: to create a decision aid that improves the knowledge of LDCT screening, decreases decisional conflict, and improves concordance of screening preferences between the official recommendations and the individual (i.e., to assist individuals with informed decision-making about whether or not to screen). Concordance with official recommendations is important, because this will ensure that the resulting population of screened individuals is consistent with that for which lung cancer screening is deemed effective.

Evidence shows that decision aids can improve decision quality as a result of better knowledge of options and their associated harms and benefits; decrease decisional conflict; and reduce the overuse and increase the underuse of screening options [7-10]. In particular, Jimbo and colleagues [9] focused on cancer screening and outlined recommendations for the evaluation of decision aids, the most important of which was to base the decision aid on a theoretical framework so that relevant outcomes are measured. The development of the decision aid in our study uses the Ottawa Decision Support Framework, a widely used theoretical framework that applies theories from psychology, social psychology, economics, and social support [11].

Whereas numerous decision aids exist for prostate, colon, and breast cancers, there are only a handful of tools that fulfill the functions of a patient decision aid, either partially or fully, with regard to LDCT screening [12-16]. We are aware of only one that has formally evaluated measures of the effect of decision aids established in the theories of behavioral research in a peer-reviewed journal [16]. However, this decision aid [16] is in the format of a video that provides average risks and benefits of LDCT screening. We know that risk varies greatly among smokers [17]; therefore, in our study, we propose to develop an alternative format: a Web-based, interactive decision aid that takes into account a more accurate depiction of an individual’s personal lung cancer risk. The decision aid will comply with the qualifying and certification criteria set out in the International Patient Decision Aid Standards instrument (IPDASi) version 4.0 [18]. The rationale for a Web-based tool is that the Web is increasingly becoming an important source of cancer information [19]. Another advantage of the Web-based format is that the content of decision aids could easily be expanded and customized for different audiences. Studies have also confirmed the feasibility of using the Web to deliver the Web-based decision aids [20].

## Methods

### Prototype Development

A prototype was developed based on the most recent clinical guidelines provided by the USPSTF [5], the IPDASi version 4.0 checklist [18], and recommendations in terms of risk communication [21-23]. The decision aid includes information on personalized lung cancer incidence risk calculated by using an established risk model [24], risk factors of lung cancer reported in the literature, harms and benefits of LDCT screening, and an explicit values clarification exercise. We also used a currently existent print-based decision aid developed by the Veterans Affairs Healthcare System as a reference [13,14].

The distinguishing factor of the Web-based version is that it allows individuals to compute their individualized lung cancer risk using an established risk model. Although all cancer screening is based on some risk factor identification, for the most common evidence-based cancer screening (e.g., colorectal, breast, and cervical cancer screening), risk factor identification involves little more than noting the relevant age and sex data. Lung cancer screening differs in very important ways in that proper application of current recommendations involves measuring risk by also identifying the individual’s cumulative tobacco exposure measured in pack-years and the timing of tobacco use. Whereas smoking history accurately identifies risk on a population basis, it is less useful for individuals because there is such great variability of lung cancer risk, even among smokers with similar exposure. Current models that more accurately quantify individual lung cancer risk incorporate up to 10 clinical and demographic variables that provide a more accurate, though complex, determination of risk. This requires sophisticated lung cancer risk prediction models [24].

In addition to accurately characterizing individual lung cancer risk, there is evidence that individuals may prefer to have such tailored information, which, in turn, may affect health-care-seeking decisions [10,25]. To this end, personalized risk in absolute terms (as a percentage) and individualized benefits in the form of icon arrays are generated by the decision aid. We also provide average harms as an estimation of individual harms (see Multimedia Appendix 1 for the current iteration of the results page).

We believe that putting LDCT screening in context with other common screening recommendations in terms of reduction of disease-specific mortality (i.e., reduction of breast cancer death by screening with mammography) will allow the individual to gauge how LDCT screening compares to other widely accepted screening practices. This has not been done with the available decision aids for LDCT lung cancer screening [13,14,16].

### Participants

Our target population comprised of potential users of LDCT screening. The specific inclusion criteria are given in Textbox 1.

These criteria applied to all 3 phases of the study detailed below: Phase I alpha testing focus groups, Phase IIa beta testing before-after survey, and Phase IIb beta testing postsurvey focus groups.

**Inclusion criteria.**

- Current and former smokers
- Aged 45-80 years
- No history of lung cancer
- No previous chest computed tomographic scan in the past year

### Recruitment Procedure

A combination of passive and active recruitment was done to form 2 convenience samples of participants: 10 for alpha testing and 60 for beta testing. An advertisement was placed on the University of Michigan’s (UM’s) online portal for volunteers of clinical studies [26], along with flyers at all of the clinics in the University of Michigan Health System (UMHS) and district libraries in Ann Arbor. Active recruitment involved in-person recruitment at a pulmonary clinic and general internal medicine clinic in the UMHS. Also, a list of participants and their contact details was generated by the Honest Broker Office of UM Medical School from medical records who fulfilled the inclusion criteria as listed above. The project coordinator verified the eligibility of all potential participants over the phone.

### Phase 1: Alpha Testing

#### Overview

We solicited feedback from public health and health risk communication experts with regard to the content and wording and how risk is expressed in the prototype. After incorporating their suggestions, we conducted focus groups with potential users of LDCT screening to test the usability of the prototype as part of the decision tool’s iterative development process. Specifically, we pretested our tool for comprehension of the content, as well as the acceptability of the design, layout, and messages conveyed. A total of 10 people participated in 1 of 2 focus groups. The eligible and willing participants were sent a Web link to work through the Web-based decision aid prototype via e-mail. All participants were asked to participate in the focus group within the week they reviewed the decision aid. Version 1 was the product of alpha testing.

#### Data Collection

The focus group was conducted by a trained external facilitator and a study team member using an interview guide developed by study team members with expertise in qualitative research and lung cancer screening decision-making (see Multimedia Appendix 2). Focus groups were held at a venue at the UM School of Public Health. At the focus group, individuals were asked to complete a survey to document the demographic makeup of the focus group participants (see Multimedia Appendix 3). Thereafter, all participants accessed the decision aid again on an iPad individually to refresh their impression. After all participants in the focus group were done reviewing the tool, the external facilitator asked questions pertaining to the usability of the decision aid. All sessions were audiotaped, and field notes were taken. Ten minutes before the scheduled end of the focus group, the participants were asked to fill out an exit survey with 4 questions (see Multimedia Appendix 4).

#### Analysis

Audio recordings were transcribed verbatim. Data analysis took place simultaneously with data collection, which, in turn, assisted in the iterative development of both the interview guide and decision aid. Using the transcriptions and field notes, a brief report was given by the study team member present at the focus groups to the rest of the study team. The study team then decided what changes to incorporate into the decision aid, forming version 1.

### Phase IIa: Beta Testing: Self-Administered Electronic Survey

#### Overview

Following alpha testing, we conducted a pilot study of version 1 of our decision aid with 60 individuals using a quasi-experimental design: a before-after study. This study design is consistent with the development of a decision aid and is an accepted method to test the effectiveness of decision aid tools under “real-life” conditions [16,27,28]. The before-after study will be followed by focus groups to gain further insight into the outcomes of interest and other spontaneous feedback about the tool in general.

#### Data Collection

Following a successful screen for eligibility, a participant was invited to come to UM to complete a series of surveys administered on a computer by Qualtrics, an online survey tool. In particular, a participant began with the knowledge survey (see Multimedia Appendix 5). This was followed by the pretest demographic, lung cancer risk, and prior screening experiences survey (see Multimedia Appendix 6); risk perception of lung cancer and lung cancer screening (see Multimedia Appendix 7); and the decision conflict scale survey (see Multimedia Appendix 8). This formed the “before” survey, and the participant was automatically redirected to the website upon clicking “submit.” The participant was instructed to explore the website for 10-15 minutes and on the final page, he or she would click on a link at the bottom that would redirect them to the “after” survey. The “after” survey comprised the knowledge, risk perception of lung cancer, and lung cancer screening; the values clarification questionnaire (see Multimedia Appendix 9); the decision conflict scale; and the acceptability survey (see Multimedia Appendix 10). The whole process lasted approximately 45 minutes.

#### Analysis

The outcomes measured were adapted from the Ottawa Decision Support Framework: knowledge of the benefits and risks of lung cancer screening, acceptability [29], decisional conflict [30], and concordance between the USPSTF’s recommendation and an individual’s preference. Concordance is a binary variable defined as “Yes” = 1, where an individual prefers to get screened (or not to get screened) and is eligible (or ineligible) and “No” = 0 if an individual prefers not to get screened (or to get screened) but is eligible (or ineligible). We also measured a participant’s risk perception as recommended by relevant literature [9]. To compare before-after outcomes, we will use the Wilcoxon signed-rank test for continuous outcomes (knowledge and decisional conflict) and McNemar’s test for binary outcomes (concordance and risk perception indicators). Frequency statistics will be computed for the acceptability items, as these will be measured only in the “after” survey. The software Stata 13 will be used to carry out all analyses [31].

### Phase IIb: Beta Testing: Focus Group

#### Overview

Following completion of the survey, participants were asked if they would like to participate in a focus group to allow them to give the study team more feedback about the decision aid they just viewed. If the answer was affirmative, the participant was told that he or she could be contacted within the month to make an appointment. They were also asked whether the decision aid had indicated that they were eligible for screening (based on USPSTF guidelines) and what the risk of dying due to lung cancer was as computed by the calculator.

Focus groups will be stratified by self-reported eligibility for LDCT screening. Given that one of the main goals of the tool is to increase concordance with USPSTF eligibility guidelines, it will be useful to have specific focus groups consisting of screen-(in)eligible individuals entirely. The aim is to have 4-8 individuals per focus group. Where possible, focus groups will also be stratified by sex and age, given eligibility for LDCT screening.

#### Data Collection

The same steps will be followed for beta testing as those used for the focus groups conducted for alpha testing. The current version of the interview guide can be seen in Multimedia Appendix 11.

#### Analysis

Thematic analysis will be done on the data yielded from the focus group sessions. Two study team members with qualitative analysis expertise will code the data separately and compare codes to establish the themes to be explored. A report will be given to the rest of the team, and all members will decide what changes to include for the next iteration of the decision aid.

### Research Ethics

All study participants will be asked to complete consent forms. Specific consent for audiotaping of focus groups will also be sought. This study was approved by the University of Michigan Medical School Institutional Review Board on 2014 June 18 (Study ID: HUM00088232).

## Results

Alpha testing is completed. Beta testing is currently being carried out. As of 2014 December 7, 60 participants had completed the before-after study. We expect to have results by 2015 January 31. Qualitative data collection and analysis are expected to be completed by 2015 May 31. The current iteration of the results page with personalized risk generated by the decision aid can be seen at Multimedia Appendix 1.

## Discussion

Preliminary results from the before-after study indicate that the decision aid improves knowledge about lung cancer screening, decreases decisional conflict, and increases concordance between USPSTF recommendations and the screening option preferred by the user. Therefore, we anticipate that the decision aid will be helpful to individuals in making informed decisions about lung cancer screening.

## Multimedia Appendix 1

An example of the current iteration of the results page.

## Multimedia Appendix 2

Focus group interview guide - alpha testing.

## Multimedia Appendix 3

Focus group participant survey.

## Multimedia Appendix 4

Focus group exit survey.

## Multimedia Appendix 5

Before-after survey: knowledge.

## Multimedia Appendix 6

Before-after survey: demographics and risk factors.

## Multimedia Appendix 7

Before-after survey: risk perception.

## Multimedia Appendix 8

Before-after survey: decisional conflict.

## Multimedia Appendix 9

Before-after survey: values clarification.

## Multimedia Appendix 10

Before-after survey: acceptability.

## Multimedia Appendix 11

Focus group interview guide – revised for beta testing.

## Abbreviations

IPDASi: International Patient Decision Aid Standards instrument
LDCT: low-dose computed tomography
NLST: National Lung Screening Trial
UM: University of Michigan
UMHS: University of Michigan Health System
USPSTF: US Preventive Services Task Force

## References

[1] American Cancer Society (2013). Cancer facts & figures 2013.

[2] Church TR, Black WC, Aberle DR, Berg CD, Clingan KL, Duan F, Fagerstrom RM, Gareen IF, Gierada DS, Jones GC, Mahon I, Marcus PM, Sicks JD, Jain A, Baum S, National Lung Screening Trial Research Team (2013). Results of initial low-dose computed tomographic screening for lung cancer. N Engl J Med.

[3] Moyer VA, US Preventive Services Task Force (2014). Screening for lung cancer: U.S. Preventive Services Task Force recommendation statement. Ann Intern Med.

[4] de Koning HJ, Meza R, Plevritis SK, ten Haaf K, Munshi VN, Jeon J, Erdogan SA, Kong CY, Han SS, van Rosmalen J, Choi SE, Pinsky PF, Berrington de Gonzalez A, Berg CD, Black WC, Tammemägi MC, Hazelton WD, Feuer EJ, McMahon PM (2014). Benefits and harms of computed tomography lung cancer screening strategies: a comparative modeling study for the U.S. Preventive Services Task Force. Ann Intern Med.

[5] Sheridan SL, Harris RP, Woolf SH, Shared Decision-Making Workgroup of the US Preventive Services Task Force (2004). Shared decision making about screening and chemoprevention: a suggested approach from the U.S. Preventive Services Task Force. Am J Prev Med.

[6] Elwyn G, O'Connor A, Stacey D, Volk R, Edwards A, Coulter A, Thomson R, Barratt A, Barry M, Bernstein S, Butow P, Clarke A, Entwistle V, Feldman-Stewart D, Holmes-Rovner M, Llewellyn-Thomas H, Moumjid N, Mulley A, Ruland C, Sepucha K, Sykes A, Whelan T, International Patient Decision Aids Standards (IPDAS) Collaboration (2006). Developing a quality criteria framework for patient decision aids: online international Delphi consensus process. BMJ.

[7] O'Connor AM, Bennett C, Stacey D, Barry MJ, Col NF, Eden KB, Entwistle V, Fiset V, Holmes-Rovner M, Khangura S, Llewellyn-Thomas H, Rovner DR (2007). Do patient decision aids meet effectiveness criteria of the international patient decision aid standards collaboration? A systematic review and meta-analysis. Med Decis Making.

[8] Stacey D, Samant R, Bennett C (2008). Decision making in oncology: a review of patient decision aids to support patient participation. CA Cancer J Clin.

[9] Jimbo M, Rana GK, Hawley S, Holmes-Rovner M, Kelly-Blake K, Nease DE, Ruffin MT (2013). What is lacking in current decision aids on cancer screening?. CA Cancer J Clin.

[10] Stacey D, Légaré F, Col NF, Bennett CL, Barry MJ, Eden KB, Holmes-Rovner M, Llewellyn-Thomas H, Lyddiatt A, Thomson R, Trevena L, Wu JH (2014). Decision aids for people facing health treatment or screening decisions. Cochrane Database Syst Rev.

[11] O’Connor AM, Stacey D, Jacobsen MJ (2011). Ottawa Hospital Research Institute.

[12] Memorial Sloan Kettering Cancer Center.

[13] Veterans Health Administration My lung cancer screening did not show lung cancer: now what?.

[14] Veterans Health Administration Screening for lung cancer pamphlet.

[15] Chen Y, Marcus MW, Niaz A, Duffy SW, Field JK (2014). MyLungRisk: a user-friendly, web-based calculator for risk assessment of lung cancer based on the validated Liverpool Lung Project risk prediction model. Int J Health Promot Educ.

[16] Volk RJ, Linder SK, Leal VB, Rabius V, Cinciripini PM, Kamath GR, Munden RF, Bevers TB (2014). Feasibility of a patient decision aid about lung cancer screening with low-dose computed tomography. Prev Med.

[17] Bach PB, Kattan MW, Thornquist MD, Kris MG, Tate RC, Barnett MJ, Hsieh LJ, Begg CB (2003). Variations in lung cancer risk among smokers. J Natl Cancer Inst.

[18] Joseph-Williams N, Newcombe R, Politi M, Durand MA, Sivell S, Stacey D, O'Connor A, Volk RJ, Edwards A, Bennett C, Pignone M, Thomson R, Elwyn G (2014). Toward minimum standards for certifying patient decision aids: a modified Delphi consensus process. Med Decis Making.

[19] Viswanath K (2005). Science and society: the communications revolution and cancer control. Nat Rev Cancer.

[20] Dorfman CS, Williams RM, Kassan EC, Red SN, Dawson DL, Tuong W, Parker ER, Ohene-Frempong J, Davis KM, Krist AH, Woolf SH, Schwartz MD, Fishman MB, Cole C, Taylor KL (2010). The development of a web- and a print-based decision aid for prostate cancer screening. BMC Med Inform Decis Mak.

[21] Fagerlin A, Zikmund-Fisher BJ, Ubel PA (2011). Helping patients decide: ten steps to better risk communication. J Natl Cancer Inst.

[22] Zikmund-Fisher BJ, Witteman HO, Dickson M, Fuhrel-Forbis A, Kahn VC, Exe NL, Valerio M, Holtzman LG, Scherer LD, Fagerlin A (2014). Blocks, ovals, or people? Icon type affects risk perceptions and recall of pictographs. Med Decis Making.

[23] Trevena LJ, Zikmund-Fisher BJ, Edwards A, Gaissmaier W, Galesic M, Han PK, King J, Lawson ML, Linder SK, Lipkus I, Ozanne E, Peters E, Timmermans D, Woloshin S (2013). Presenting quantitative information about decision outcomes: a risk communication primer for patient decision aid developers. BMC Med Inform Decis Mak.

[24] Tammemägi MC, Katki HA, Hocking WG, Church TR, Caporaso N, Kvale PA, Chaturvedi AK, Silvestri GA, Riley TL, Commins J, Berg CD (2013). Selection criteria for lung-cancer screening. N Engl J Med.

[25] Holbrook AM, Pullenayegum EM, Troyan SM, Nikitovic M, Crowther MA (2013). Personalized benefit-harm information influences patient decisions regarding warfarin. J Popul Ther Clin Pharmacol.

[26] University of Michigan Clinical Studies.

[27] Coulter A, Stilwell D, Kryworuchko J, Mullen PD, Ng CJ, van der Weijden T (2013). A systematic development process for patient decision aids. BMC Med Inform Decis Mak.

[28] Schonberg MA, Hamel MB, Davis RB, Griggs MC, Wee CC, Fagerlin A, Marcantonio ER (2014). Development and evaluation of a decision aid on mammography screening for women 75 years and older. JAMA Intern Med.

[29] O’Connor AM, Cranney A (1996). User manual – acceptability [modified 2002].

[30] O’Connor AM (1993). Decisional conflict scale [updated 2010].

[31] StataCorp (2013). STATA 13.

