# Supplementary figures and images for: Development and Validation of a Personalized, Web-Based Decision Aid for Lung Cancer Screening Using Mixed Methods: A Study Protocol
Source: JMIR Res Protoc. 2014 Dec 19;3(4):e78. doi: 10.2196/resprot.4039 (PMC4376198; doi:10.2196/resprot.4039)

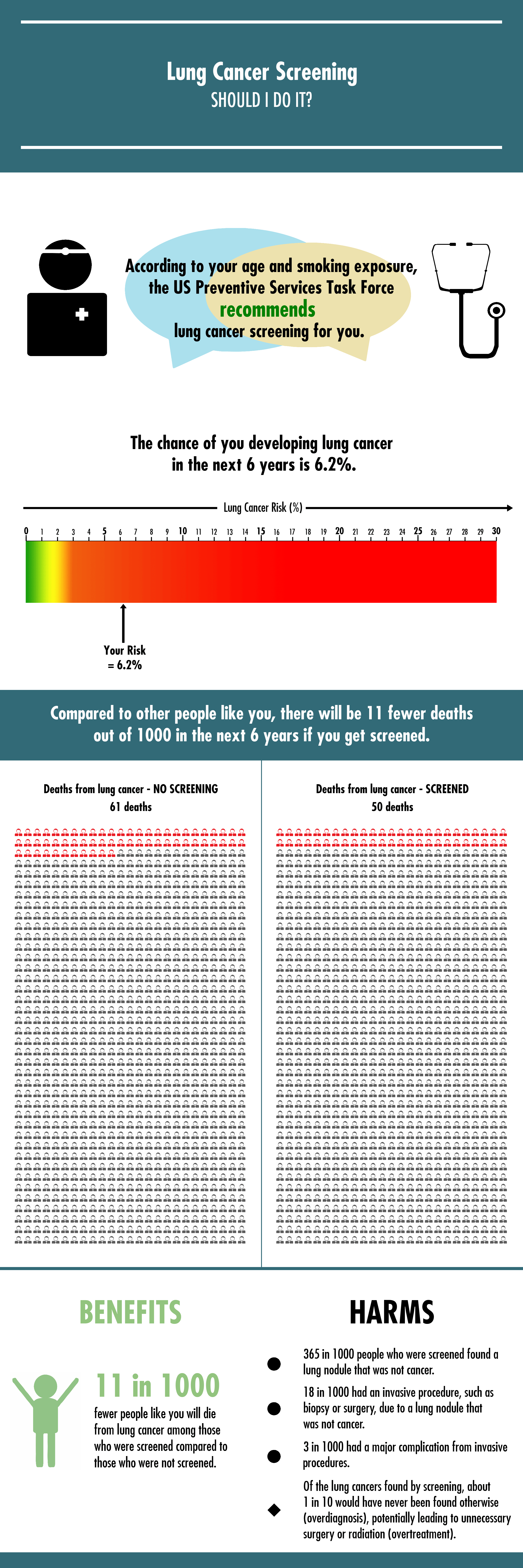

Supplement: Supplementary file 1 [file resprot_v3i4e78_app1.png]
