# Supplementary material for: Development and Validation of a Personalized, Web-Based Decision Aid for Lung Cancer Screening Using Mixed Methods: A Study Protocol
Source: JMIR Res Protoc. 2014 Dec 19;3(4):e78. doi: 10.2196/resprot.4039 (PMC4376198; doi:10.2196/resprot.4039)
Supplement: Supplementary file 2 [file resprot_v3i4e78_app2.pdf]

Focus group decision guide: Lung cancer CT screening decision aid tool.

Question	Probe	Notes
Q1. I am going to start off by asking you how you felt about the presentation of the tool/website.	- Do you like the layout? Was it easy to navigate between the different pages?<br>- Did you need help navigating from someone?<br>- Do you like the design? Was it attractive/appealing? Did it “look good” or did it put you off? Why or why not?	
Q2. Now I am going to ask you about the information about lung cancer screening. Did you find it easy to understand?	- Was it easy/confusing/too detailed/too little detail?<br>- Did you understand the benefits/up side of screening?<br>- Did you understand the risks/down side of screening?<br>- What other information would you have liked?	
Q3. I would like to ask you the same questions for the way information was presented on lung cancer itself.	- Was it easy/confusing/too detailed/too little detail?<br>- From the material, do you understand what causes lung cancer?<br>- And did you understand what can help you decrease your chances of developing lung cancer?<br>- What other information would you have liked?	
Q4. What did you like about the tool?	- Did it help you clarify some questions?	

	- Did you learn something new? What did you learn that you did not know?<br>- Did you learn something new to help you make a decision?	
Q5. What did you not like about the tool?	- Did it create more questions?<br>- Did it cause more confusion?	
Q6. Did you find the tool relevant?	- Did you learn something new to help you come to a decision?<br>- Would you consider talking to a doctor about lung cancer CT screening?	
Q7. For those who accessed the tool online, where did you do it?	- Did you do this at home or at work?<br>- Did you do it on your phone, on a tablet, or a laptop or computer?	
Q8. Is there anything else that you would like to add that we have not covered?		
