# Supplementary material for: Development and Validation of a Personalized, Web-Based Decision Aid for Lung Cancer Screening Using Mixed Methods: A Study Protocol
Source: JMIR Res Protoc. 2014 Dec 19;3(4):e78. doi: 10.2196/resprot.4039 (PMC4376198; doi:10.2196/resprot.4039)
Supplement: Supplementary file 3 [file resprot_v3i4e78_app3.pdf]

Focus Group Participant Survey

Please answer the following questions before we get started with the group. These are just to help us get a better idea of who is here today. You do not need to put your name on this form and you do not need to answer any questions you do not feel comfortable with.

1. **What is your gender? (Please check the option that applies)**
   - ☐ Male
   - ☐ Female

2. **What is your age?**

   ______ years old.

3. **What is the highest grade or year of school you completed?**
   - ☐ Less than high school
   - ☐ High school graduate
   - ☐ Some training after high school
   - ☐ Some college
   - ☐ College graduate
   - ☐ Postgraduate or professional degree

4. **How would you describe your race/ethnicity? (Check all that apply)**
   - ☐ African or Black American
   - ☐ American Indian/Alaskan Native
   - ☐ Asian
   - ☐ Hispanic
   - ☐ Indian
   - ☐ White
   - ☐ Other (specify): ___________________

5. **What is your height?**

   _______ ft. ______ in.

6. **What is your weight?**

   _______ lbs.

7. At what age did you start smoking cigarettes?

______

8. Do you smoke cigarettes now?

- Yes → Skip to question 10.
- No

9. At what age did you quit smoking for the last time?

______ years old

10. For how many years total have you smoked cigarettes?

______ years

11. On average, how many cigarettes do/did your smoke per day?

______

12. Have you ever been told by a doctor that you have cancer?

- Yes
- No
- Don’t know/Not sure

13. Does your family have a history of lung cancer?

- Yes
- No
- Don’t know/Not sure

14. Have you ever been told by a doctor that you have obstructive pulmonary disease (COPD)?

- Yes
- No
- Don’t know/Not sure

15. Do you have internet access at home and/or at work?

- Yes, but at home only
- Yes, but at work only
- Yes, I have internet at home and at work
- No, I do not have internet at home or at work
- Don’t know/Not sure

16. From whom/where did you first hear about lung cancer CT screening?

- Health care professional
- Family
- Friends
- Internet
- Newspaper/magazine
- Social media
- The decision aid tool from this study
- Other (specify): ___________________
- Don’t know/Not sure

17. Has a doctor or health care provider ever suggested you undergo lung cancer screening?

- Yes
- No
- Don’t know/Not sure

18. Have you ever been screened for any other diseases?

- Yes
- No → Skip to question 20.
- Don’t know/Not sure → Skip to question 20.

19. What have you been screened for?

- Diabetes
- Other types of cancer
- Cholesterol
- Other (specify): _________________
- Don’t know/Not sure

20. How would you describe your approach to health care? Check the one that most applies to you.

- I prefer to “watch and wait,” and often go by the saying “if it ain’t broke, don’t fix it.”
- I like doing things that could fix a health problem such as getting tests or taking medicine. I do not like watching and waiting for my body to fix itself.
- I am somewhere in between the first and second statement.
- Don’t know/Not sure

21. What was your approximate household income last year?

- Less than $15000
- $15000 to $24999
- $25000 to $34999
- $35000 to $44999
- $45000 to $54999
- $55000 or more
- Don’t know/Not sure
- Prefer not to answer
