# Supplementary material for: Development and Validation of a Personalized, Web-Based Decision Aid for Lung Cancer Screening Using Mixed Methods: A Study Protocol
Source: JMIR Res Protoc. 2014 Dec 19;3(4):e78. doi: 10.2196/resprot.4039 (PMC4376198; doi:10.2196/resprot.4039)
Supplement: Supplementary file 4 [file resprot_v3i4e78_app4.pdf]

Focus group – Exit survey

1. Overall, how satisfied are you with the decision tool? (circle one)

   1. Not at all satisfied
   2. Slightly satisfied
   3. Moderately satisfied
   4. Very satisfied
   5. Extremely satisfied

2. How difficult or easy will it be to use this decision tool at home? (circle one)

   1. Very difficult
   2. Difficult
   3. Neutral
   4. Easy
   5. Very easy

3. Rate how much you agree or disagree with the following statement:

"This tool will help people think about what is most important in making a lung cancer screening decision." (circle one)

   1. Strongly disagree
   2. Disagree
   3. Neither agree nor disagree
   4. Agree
   5. Strongly agree

4) Rate how much you agree or disagree with the following statement:

“The tool will help people in making a decision about lung cancer screening that is right for them.” (circle one)

1. Strongly disagree
2. Disagree
3. Neither agree nor disagree
4. Agree
5. Strongly agree
