# Supplementary material for: Development and Validation of a Personalized, Web-Based Decision Aid for Lung Cancer Screening Using Mixed Methods: A Study Protocol
Source: JMIR Res Protoc. 2014 Dec 19;3(4):e78. doi: 10.2196/resprot.4039 (PMC4376198; doi:10.2196/resprot.4039)
Supplement: Supplementary file 5 [file resprot_v3i4e78_app5.pdf]

Knowledge of risks and benefits of lung cancer screening[1]

What I know about lung cancer and lung cancer screening with low-dose computed tomography now

Here are some questions about lung cancer screening. We would like to know how familiar you are with lung cancer screening before you use the decision aid.

Below are some statements about lung cancer screening. Please show whether you think they are true, false, or you are not sure by selecting one of these options.

1. **Factors that increase chances of developing lung cancer include:**

Smoking	True	False	Unsure
Family history of lung cancer	True	False	Unsure
Getting the flu	True	False	Unsure
Chronic obstructive pulmonary disease (COPD)	True	False	Unsure
Age	True	False	Unsure
Drinking alcohol	True	False	Unsure

2. **Possible benefits of lung cancer screening are:**

It can cure cancer	True	False	Unsure
It lowers your chances of dying from lung cancer	True	False	Unsure
It lowers your chances of developing lung nodules	True	False	Unsure

3. **Possible harms of lung cancer screening include:**

You may find some things in your lungs that are not cancer but would need to an extra test to check	True	False	Unsure
You may need to get an extra test which can cause complications	True	False	Unsure
There are no harms associated with screening	True	False	Unsure

4. **Indicate whether the following individuals would be eligible for screening based on their age. Select yes for eligible, no for ineligible, or unsure. They all meet the smoking status and pack-year criteria:**

[1] This will be administered before and after participants view the tool. If a respondent correctly selects “true” or “false”, a score value of 1 will be given, and 0 for incorrect responses or unsure.

Age: 45 years old	True	False	Unsure
Age: 55 years old	True	False	Unsure
Age: 60 years old	True	False	Unsure
Age: 95 years old	True	False	Unsure

5. What percentage of lumps found on your lung by the CT screening is NOT going to be cancer?

- o More than 90%
- o 50 – 60%
- o 10 – 20%
- o Less than 5%
- o Don't know/Not sure
