# Supplementary material for: Development and Validation of a Personalized, Web-Based Decision Aid for Lung Cancer Screening Using Mixed Methods: A Study Protocol
Source: JMIR Res Protoc. 2014 Dec 19;3(4):e78. doi: 10.2196/resprot.4039 (PMC4376198; doi:10.2196/resprot.4039)
Supplement: Supplementary file 6 [file resprot_v3i4e78_app6.pdf]

Pre-test demographic, lung cancer risk and prior screening experiences survey[i]

1. Please state your year of birth.
   ____

2. What is the highest grade or year of school you completed?

   1 Less than high school
   2 High school graduate
   3 Some training after high school
   4 Some college
   5 College graduate
   6 Postgraduate or professional degree

3. Which one or more of the following would you say is your race? [Check all that apply]

   1 African or Black American
   2 American Indian/Alaskan Native
   3 Asian
   4 Hispanic
   5 Indian
   6 White
   7 Other
   99 Refused

4. What is your height?
_____ ft. ____ in.

5. What is your weight?
_________ lbs.

6. At what age did you start smoking cigarettes?
____

7. Do you smoke cigarettes now?
   1 Yes (skip to Q. 10)
   2 No

8. At what age did you quit smoking for the last time?
____

9. For how many years total have you smoked cigarettes?
_____

10. On average, how many cigarettes do/did you smoke per day?
_____

11. Have you ever been told by a doctor that you have cancer?

1 Yes
2 No
8 Don’t know/Not sure
9 Refused

12. Does your family have a history of cancer?

1 Yes
2 No
8 Don’t know/Not sure
9 Refused

13. Have you ever been told by a doctor that you have chronic obstructive pulmonary disease (COPD)?

1 Yes
2 No
3 Don’t know/Not sure
4 Refused

14. Low-dose computed tomography (CT) screening is used to detect lung cancer. Have you read or heard about this type of lung cancer screening?

1 Yes
2 No [Skip to Q.16]
8 Don’t know/Not sure [Skip to Q.16]
9 Refused [Skip to Q.16]

15. From whom/where did you hear about lung cancer CT screening? [Mark all that apply]

1 Health care professional
2 Family
3 Friends/acquaintances
4 Internet
5 Newspaper/magazine
6 Social media
7 Other, please specify: ________
8 Don’t know/Not sure
9 Refused

16. Do you have internet access at home/work?

1 Yes
2 No
8 Don’t know/Not sure
9 Refused

17. Has a doctor or other health care provider ever suggested you undergo lung cancer screening?

1 Yes
2 No
8 Don’t know/Not sure
9 Refused

18. Have you ever been screened for any other diseases?

1 Yes
2 No [Skip to Q.20]
8 Don’t know/Not sure [Skip to Q.20]
9 Refused [Skip to Q.20]

19. What have you been screened for?

1 Diabetes
2 Other types of cancer
3 Cholesterol
4 Other. Please specify: _______________
8 Don’t know/Not sure
9 Refused

20. In general, would you say your health is:

1 Excellent
2 Very good
3 Good
4 Fair
5 Poor

21. How often do you have someone (like a family member, friend, hospital/clinic worker, or caregiver) help you read hospital materials?

1 Always
2 Often
3 Sometimes
4 Occasionally
5 Never

22. How confident are you filling out medical forms by yourself?

1 Extremely
2 Quite a bit
3 Somewhat
4 A little bit
5 Not at all

23. How often do you have problems learning about your medical condition because of difficulty understanding written information?

1 Always
2 Often
3 Sometimes
4 Occasionally
5 Never

24. How often do you find numerical information to be useful?

1 Never
2 Rarely
3 Sometimes
4 Fairly often
5 Very often

---

[i] Questions 1 to 13 will be used as input to risk model to calculate personalized risk and benefits for lung cancer screening.
