# Supplementary material for: Development and Validation of a Personalized, Web-Based Decision Aid for Lung Cancer Screening Using Mixed Methods: A Study Protocol
Source: JMIR Res Protoc. 2014 Dec 19;3(4):e78. doi: 10.2196/resprot.4039 (PMC4376198; doi:10.2196/resprot.4039)
Supplement: Supplementary file 7 [file resprot_v3i4e78_app7.pdf]

Risk Perception of lung cancer and lung cancer screening

1. In your opinion, how *likely* are you to die from cancer without CT screening? (10-point scale from “Not at all likely” to “Extremely likely”)

2. How *worried* would you be about dying from cancer without CT screening? (10-point scale from “Not at all worried” to “Extremely worried”)

3. In your opinion, how *effective* is CT screening at reducing your chance of dying from cancer? (10-point scale from “Not at all effective” to “Extremely effective”)

4. If you were to get CT screening, how *likely* are you to avoid a cancer death? (10-point scale from “Not at all likely” to “Extremely likely”)

5. If you were to get CT screening, how *likely* are you to experience a harm from screening? (10-point scale from “Not at all likely” to “Extremely likely”)

6. If you were to get CT screening, how *worried* would you be about experiencing a harm from screening? (10-point scale from “Not at all worried” to “Extremely worried”)

7. Among people getting CT screening, how *common* do you think harms due to screening are? (10-point scale from “Not at all common” to “Extremely common”)

8. Considering both the risks and benefits CT screening, how *good of a choice* is CT screening as a way to reduce your risk of dying from cancer? (10-point scale from “Not a good choice at all” to “An extremely good choice”)

9. If getting CT screening was free for you, would you get screened? (yes/no)

10. If getting CT screening would cost you $50, would you get screened? (yes/no)

11. If getting CT screening would cost you $200, would you get screened? (yes/no)
