# Supplementary material for: Development and Validation of a Personalized, Web-Based Decision Aid for Lung Cancer Screening Using Mixed Methods: A Study Protocol
Source: JMIR Res Protoc. 2014 Dec 19;3(4):e78. doi: 10.2196/resprot.4039 (PMC4376198; doi:10.2196/resprot.4039)
Supplement: Supplementary file 8 [file resprot_v3i4e78_app8.pdf]

Decisional conflict scale[1]

My difficulty in making the decision to do lung cancer screening

A. Which option do you prefer right now in terms of lung cancer screening?

1 I prefer to screen
2 I prefer *not* to screen
3 Unsure

B. Considering the option you prefer, please answer the following questions:

1. Do you know which options are available to you?

1 Yes [0]
2 Unsure [2]
3 No [4]

2. Do you know the benefits of each option?

1 Yes [0]
2 Unsure [2]
3 No [4]

3. Do you know the risks of each option?
1 Yes [0]
2 Unsure [2]
3 No [4]

4. Are you clear about which benefits matter most to you?
1 Yes [0]
2 Unsure [2]
3 No [4]

5. Are you clear about which risks matter most to you?
1 Yes [0]
2 Unsure [2]
3 No [4]

6. Do you have enough support from others to make a choice?
1 Yes [0]
2 Unsure [2]
3 No [4]

7. Are you choosing without pressure from others?
1 Yes [0]

[1] Scores for each response are in square brackets. These scores will be summed to obtain the total decisional conflict score, where lower scores indicate lower decisional conflict.

2 Unsure [2]
3 No [4]

8. Do you have enough advice to make a choice?
   1 Yes [0]
   2 Unsure [2]
   3 No [4]

9. Are you clear about the best choice for you?
   1 Yes [0]
   2 Unsure [2]
   3 No [4]

10. Do you feel sure about what to choose?
   1 Yes [0]
   2 Unsure [2]
   3 No [4]
