# Supplementary material for: Development and Validation of a Personalized, Web-Based Decision Aid for Lung Cancer Screening Using Mixed Methods: A Study Protocol
Source: JMIR Res Protoc. 2014 Dec 19;3(4):e78. doi: 10.2196/resprot.4039 (PMC4376198; doi:10.2196/resprot.4039)
Supplement: Supplementary file 9 [file resprot_v3i4e78_app9.pdf]

Values clarification exercise: What are the pros and cons of lung cancer screening

YES: Getting screened for lung cancer. I think the **PROS** of screening are:

1. ______________________________

____

On a scale of 1 to 10, how important is this reason to you? Please click on a number that applies.

0	1	2	3	4	5	6	7	8	9	10

Not important — Very important

2. ______________________________

____

On a scale of 1 to 10, how important is this reason to you? Please click on a number that applies.

0	1	2	3	4	5	6	7	8	9	10

Not important — Very important

NO: Not getting screened for lung cancer. I think the **CONS** of screening are:

1. ______________________________

____

On a scale of 1 to 10, how important is this reason to you? Please click on a number that applies.

0	1	2	3	4	5	6	7	8	9	10

Not important — Very important

2. ______________________________

____

On a scale of 1 to 10, how important is this reason to you? Please click on a number that applies.

0	1	2	3	4	5	6	7	8	9	10

Not important — Very important
