# Supplementary material for: Development and Validation of a Personalized, Web-Based Decision Aid for Lung Cancer Screening Using Mixed Methods: A Study Protocol
Source: JMIR Res Protoc. 2014 Dec 19;3(4):e78. doi: 10.2196/resprot.4039 (PMC4376198; doi:10.2196/resprot.4039)
Supplement: Supplementary file 10 [file resprot_v3i4e78_app10.pdf]

Acceptability

<u>My thoughts on the decision aid on lung cancer screening</u>

We would like to know what you think about the decision aid you have just reviewed.

1. Please rate each section, by selecting “poor”, “fair”, “good”, or “excellent” to show what you think about the way the information was presented on:

The things that increase your chances of lung cancer	Poor	Fair	Good	Excellent
Benefits of screening	Poor	Fair	Good	Excellent
Harms of screening	Poor	Fair	Good	Excellent

2. The length of the presentation was (choose one):

    Too long
    Too short
    Just right

3. The amount of information was (choose one):

    Too much information
    Too little information
    Just right

4. I found the presentation (choose one):

    Slanted towards being screened for lung cancer
    Slanted towards not being screened for lung cancer
    Balanced

5. Did you find this decision aid useful when you were making your decision about lung cancer screening?

    Yes
    No

6. What did you think of the way that risks of lung cancer screening was presented? Was it (choose one):

    Easy to understand
    Confusing

7. Do you think we included enough information to help people decide on whether or not to be screened for lung cancer?

    Yes
    No
