# Supplementary material for: Development and Validation of a Personalized, Web-Based Decision Aid for Lung Cancer Screening Using Mixed Methods: A Study Protocol
Source: JMIR Res Protoc. 2014 Dec 19;3(4):e78. doi: 10.2196/resprot.4039 (PMC4376198; doi:10.2196/resprot.4039)
Supplement: Supplementary file 11 [file resprot_v3i4e78_app11.pdf]

Focus group question guide: Lung cancer CT screening decision aid tool.

Question	Probe	Notes
Q1. Before we get into the "nitty gritty" of the website, I am going to start off by asking you: What was your first impression of the website?	- How did you feel about the design?<br>- What about the layout and navigation?	
Q2. Now I am going to ask you a content-specific question. The information about lung cancer *screening*, how did you feel about the information that was presented?	- How did you feel the about the explanation of what lung cancer screening involves?<br>- What about the presentation of the harms and benefits of screening, how did you feel about that?<br>- And what about the comparison between lung cancer screening and mammography, what did you think about that?<br>- Were there some words or phrasing that were not straightforward to understand? If so, could you point out where?<br>- Where do you think improvements can be made (otherwise)?	
Q3. On a similar note, how did you find the information about the causes of lung cancer?	- How did you feel the about the explanation of what causes lung cancer?<br>- What about the page that talks about reducing risks of getting lung cancer, what did you think about that information?	

	- What other information would you have liked? Or, where do you think improvements can be made with regards to what causes lung cancer and how to reduce the risk of getting it?	
Q4. Now I would like to draw your attention to the results page and ask you for your feedback.	- Which part drew your attention the most, and why?<br>- Which part was not clear and can you tell me more about that?<br>- What were some of your feelings when you were reading about your own numbers?<br>- Show the single panel vs double panel of little men and ask for feedback and preferences.	
Q5. Overall, what did you like about the website?	- Could you tell me, perhaps, if it helped you clarify some questions? And what were they?<br>- Did you learn something new? What did you learn that you did not know?<br>- Anything else that you liked? It could be anything from colour, font size, design, the navigation.	
Q6. Similarly, what did you not like about the website?	- For example, while you learnt something new, did it also create more questions, or confusion?<br>- Anything else you did **not** like? Color, font size, navigation?	
Q7. Would you use this website yourself? Would you recommend others to use it? Tell me more.	- For example, would you consider talking to a doctor about lung cancer CT screening?	

Q8. For those who accessed the website online, where did you do it?	- Did you do this at home or at work?<br>- Did you do it on your phone, on a tablet, or a laptop or computer?	
Q9. Is there anything else that you would like to add that we have not covered?<br><br>If you had one minute to give advice to the designer of the website, what would you say?		
